# Carbon stocks for different land cover types in Mainland Tanzania

**DOI:** 10.1186/s13021-019-0120-1

**Published:** 2019-04-27

**Authors:** Ernest William Mauya, Wilson Ancelm Mugasha, Marco Andrew Njana, Eliakimu Zahabu, Rogers Malimbwi

**Affiliations:** 1 Department of Forest Engineering and Wood Sciences, College of Forestry, Wildlife and Tourism, Sokoine University of Agriculture, Box 3012, Morogoro, Tanzania; 2 Department of Forest Resources Assessment and Management, College of Forestry, Wildlife and Tourism, Sokoine University of Agriculture, Box 3013, Morogoro, Tanzania; 3 National Carbon Monitoring Centre, Box 3013, Morogoro, Tanzania

**Keywords:** Carbon stock, REDD+, FREL, Emission factor, Uncertainty

## Abstract

**Background:**

Developing countries participating in the mitigation mechanism of reducing emissions from deforestation and forest degradation (REDD+), are required to establish a forest reference emission level (FREL), if they wish to seek financial support to reduce carbon emissions from deforestation and forest degradation. However, establishment of FREL relies heavily on the accurate estimates of carbon stock as one of the input variable for computation of the emission factors (EFs). The product of an EF and activity data, such as the area of deforestation, results in the total emissions needed for establishment of FREL. This study presents the carbon stock estimates for different land cover classes based on an analysis of Tanzania’s national forest inventory data generated through the National Forest Resources Monitoring and Assessment (NAFORMA).

**Results:**

Carbon stocks were estimated in three carbon pools, namely aboveground, belowground, and deadwood for each of the three land cover classes (i.e. Forest, non-forest, and wetland). The weighted average carbon stock was 33.35 t C ha^−1^ for forest land, 4.28 t ha^−1^ for wetland and 5.81 t ha^−1^ for non-forest land. The uncertainty values were 0.9% for forest land, 11.3% for wetland and 1.8% for non-forest land. Average carbon stocks for land cover sub-classes, which make up the above mentioned major land cover classes, are also presented in our study.

**Conclusions:**

The values presented in this paper correspond to IPCC tier 3 and can be used for carbon estimation at the national scale for the respective major primary vegetation type for various purposes including REDD+. However, if local based estimates values are needed, the use of auxiliary data to enhance the precision of the area of interest is recommended.

## Background

The significance of forests, particularly tropical forests, in the global carbon cycle has led to the consideration and recognition of forest-based climate change mitigation measures in the international climate negotiations, agreements and policy frameworks. Accordingly, to date a number of agreements have been reached [1]. Mostly notably, is the adoption of the Bali Action/roadmap in 2007 at the 13th Conference of Parties (COP13) and the subsequent recognition of REDD+ scheme, which is an initiative referring to reducing emissions from deforestation and forest degradation [2]. Essentially, REDD+ involves implementation of a variety of policy approaches and incentive plans to the activities related with reduction in deforestation and forest degradation, as well as forest conservation, sustainable management of forests and the enhancement of forest carbon stocks in the tropical forests [3]. This mechanism has been accepted as a low-cost and promising approach for mitigating climate change [4] that will also secure many ecological functions of forests, including biodiversity conservation and provision of a number of ecosystem services.

The interest among developing countries to prepare for implementation of REDD+ projects, and in testing the potential mechanisms, has increased tremendously since the initial discussions under UNFCCC in 2005. However, in order to effectively implement REDD+ at the national level, countries are required to develop four key components if they aim to undertake REDD+ activities and to be eligible for financial compensation [5]: (1) a national strategy or action plan; (2) a national forest reference emission level (FREL) and/or forest reference level (FRL); (3) a robust and transparent national forest monitoring system for Measurement, Reporting and Verification (MRV) of the REDD+ activities; and (4) a system for providing information on how the safeguards are addressed or respected. Forest reference emission level (FREL) being among the four key elements of the REDD+ is defined as the benchmark for carbon emissions against which a country’s performance in implementing REDD+ activities can be assessed and credited [1]. However, estimation of carbon emission as key variables for setting up FREL, requires information on activity data (AD) which refers to the area of forest change (in hectare), e.g., forest converted to grassland or forest converted to cropland and Emission Factors (EF) which relates to the carbon stock change estimations per unit of activity (in carbon per hectare) [6, 7]. To ensure that there is consistency in estimation of carbon emission, the Intergovernmental Panel on Climate Change (IPCC) had provided three hierarchical tiers for reporting the different levels of detail and accuracy. The Tier 1 approach employs the default emission factors provided in the IPCC Guidelines while Tier 2 approach uses country-specific emission factors. On the other hand, Tier 3 approach uses higher order methods including models and inventory measurement systems tailored to address national circumstances, repeated over time, and driven by high-resolution activity data and disaggregated at sub-national to fine grid scales [10]. The IPCC recommends using higher Tiers for the measurement of important sources and sinks. Tier 2 or 3 methods are regarded as higher tiers since they provide the desired level of accuracy for important components of the greenhouse gas (GHG) inventory. However, higher Tier methods require more data and are more expensive, because they involve monitoring of local variables [8]. This causes a challenge to many of the REDD+ countries due to the lack of the data from continuous National Forest Inventories (NFI) which can support estimation of EF using higher Tier approaches.

Mainland Tanzania, unlike many other developing countries, has wealth of up to-date NFI data that can support estimation of EF. The first NFI in Mainland Tanzania was implemented between 2009 and 2014 through National Forest Resources Monitoring and Assessment (NAFORMA) Project. The NFI covered different land cover types including different vegetation and land use types where the three IPCC carbon pools of aboveground biomass (AGB), belowground biomass (BGB) and dead wood biomass (DWB) were assessed [9]. This allows for land cover specific estimation of emissions of greenhouse gas and reduces uncertainties. This is in line with IPCC guidelines which emphasize that estimation and reporting of greenhouse gases should be complete by considering all land covers [10]. A quarter of all inventoried plots are permanent sample plots for the purpose of repeated measurement over time. Irrespective of such initiatives, this paper is the first attempt to document carbon densities for different land cover type using the NFI data. Such information is important for the ongoing REDD+ reporting activities as well as for conventional objectives related with sustainable forest management.

Furthermore, according to IPCC [10], for complete inventory of greenhouse gas emissions, uncertainties should be estimated at different spatial scales as well as for the component parts such as carbon densities, emission factors, activity data and other estimation parameters for each category. Therefore, in line with this, our study also reports the uncertainty of carbon stocks for different land cover sub-classes.

## Methods

### Study area

The United Republic of Tanzania is a union of Mainland Tanzania and Zanzibar, it is located between longitude 29° and 41° East and Latitude 1° and 12° South. Tanzania mainland is endowed with a wide range of natural resources. The country has a very diverse climate, depending on altitude and latitude. The mean annual rainfall varies from below 500 to over 2000 mm per annum. The rainfall for the large part of the country is bimodal with short rains from October to December and long rains from March to May. The main forest types include deciduous miombo woodlands in the western, central and southern parts of the country, *Acacia*-*Commiphora* woodlands in the northern regions, coastal forests and woodland mosaics in the east, mangrove forests along the coast of the Indian Ocean, and closed canopy forests, which grow on the ancient mountains of the Eastern Arc, along the Albertine Rift close to Lake Tanganyika in the west, and on the younger volcanic mountains in the north and central parts of the country [11].

**Fig. 1:**
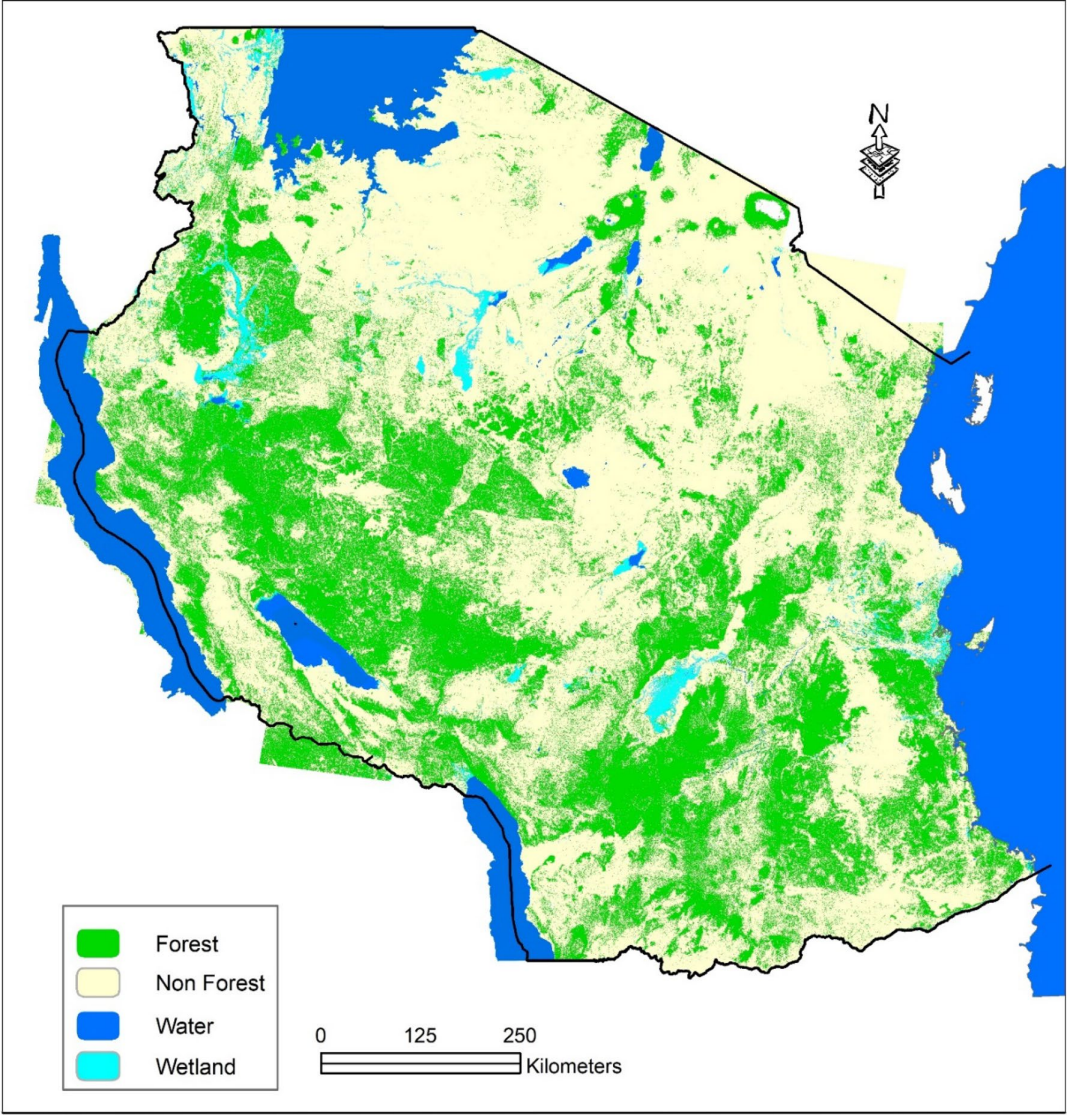
Distribution of the four primary land cover classes

Based on the recently land use land cover (LULC) change analysis for Mainland Tanzania there are four primary land cover classes of (1) forest, (2) non-forest, (3) water and (4) wetlands (Table 1 and Fig. 1). Each primary class consists of several land cover sub-classes. In this regard ‘Forest’ means an area of land with at least 0.5 ha, with a minimum tree crown cover of 10% or with existing tree species planted or natural having the potential of attaining more than 10% crown cover, and with trees which have the potential or have reached a minimum height of 3 m at maturity in situ [12].

**Table 1:** Classification of land cover types in Mainland Tanzania

Land cover sub-class	Primary class
Forest: Plantation	Forest
Forest: Mangrove	Forest
Forest: Humid montane	Forest
Forest: Lowland	Forest
Woodland: Closed (> 40%)	Forest
Woodland: Open (10–40%)	Forest
Cultivated land (Wooded crops): Mixed tree cropping	Forest
Cultivated land (Wooded crops): Wooded crops	Forest
Woodland (Wooded crops): Scattered cropland (Unspecified density)	Forest
Bushland: Thicket	Forest
Bushland: Thicket with emergent trees	Forest
Bushland: Dense	Non forest
Bushland: Emergent trees	Non forest
Bushland: Open	Non forest
Bushland: Scattered cultivation	Non forest
Cultivated land: Agro-forestry system	Non forest
Cultivated land: Grain crops	Non forest
Cultivated land: Herbaceous crops	Non forest
Grassland: Bushed	Non forest
Grassland: Open	Non forest
Grassland: Scattered cropland	Non forest
Grassland: Wooded	Non forest
N/A	Non forest
Open land: Bare soil	Non forest
Open land: Rock outcrops	Non forest
Open land: Salt crusts	Non forest
Other areas	Non forest
Water: Inland water	Wetland
Water: Swamp	Wetland

### Sampling design

**Fig. 2:**
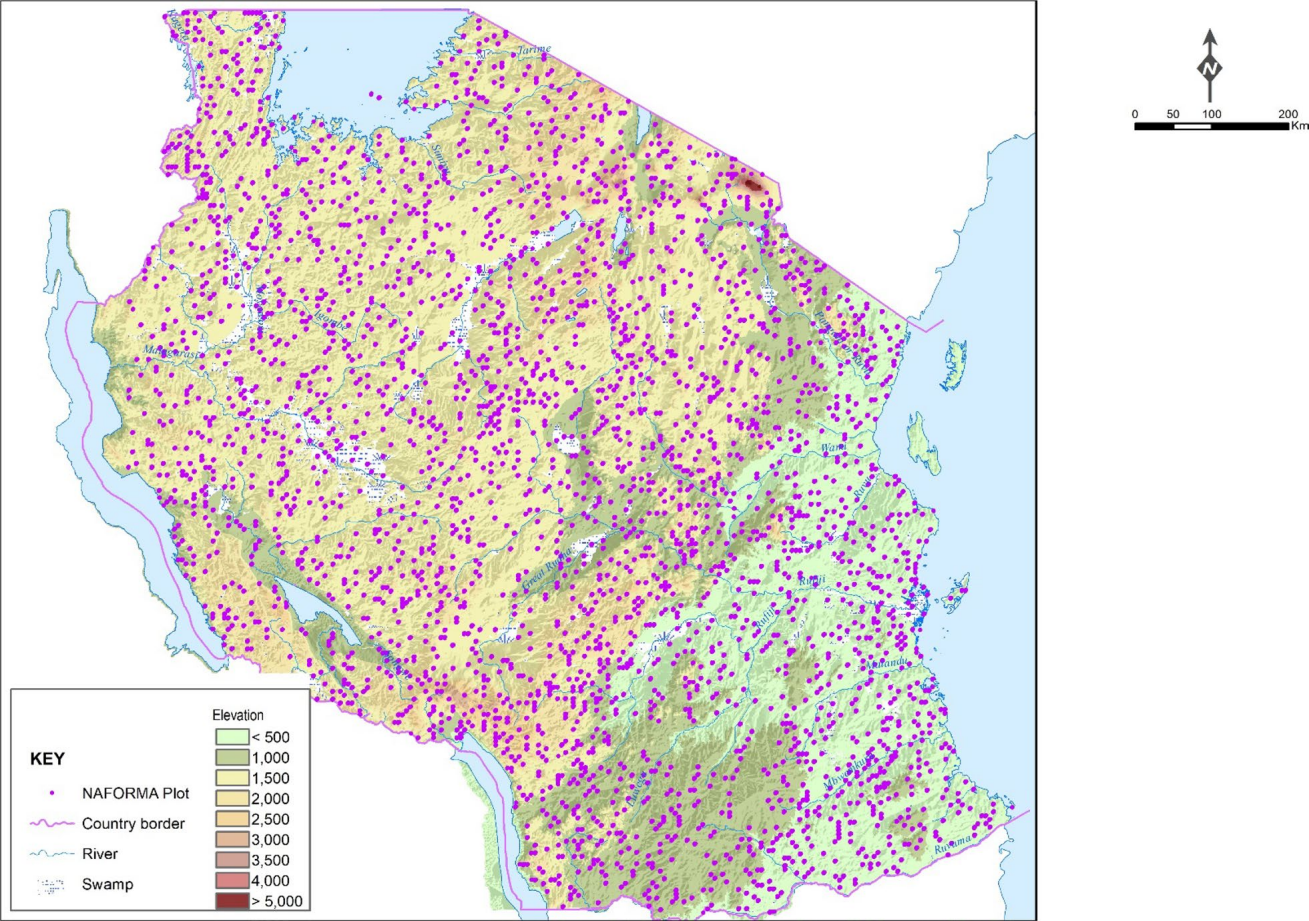
Distribution of sample plots in Mainland Tanzania

**Fig. 3:**
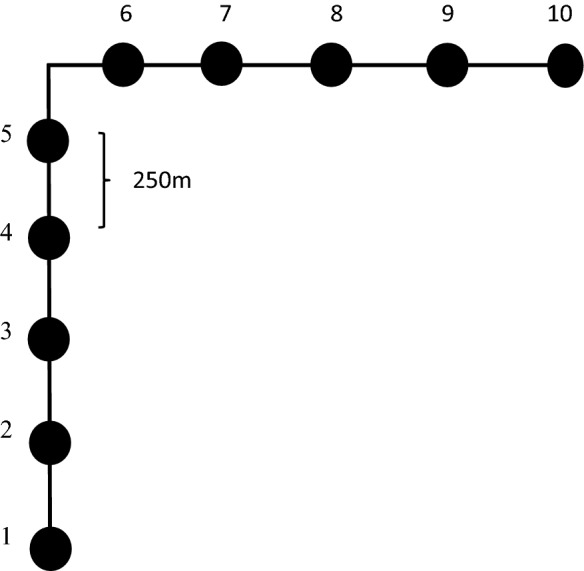
NFI cluster design (black solid circles = plot)

The data used for estimation of carbon stocks presented in this paper were based on the Mainland Tanzania NFI data which is commonly referred as NAFORMA, carried out from 2009 to 2014. The NFI was developed based on double sampling for stratification and optimal allocation of plots. The first-phase sample consists of clusters of plots laid on a 5 × 5 km grid over mainland Tanzania. The first-phase clusters were stratified based on a combination of three criteria namely; predicted growing stock, time consumption for cluster measurements and slope of the terrain. At national level, the first-phase clusters were assigned to 18 pre-defined strata according to the three criteria [see 13, 14]. Within each stratum, second-phase samples of clusters were selected using optimal allocation [15] with cost functions tailored for each stratum using a simulation approach described in Tomppo, Malimbwi, Katila, Mäkisara, Henttonen, Chamuya, Zahabu and Otieno [13]. As a result, greater sampling intensity was allocated to strata with more variation and larger predicted growing stock and less sampling intensity to strata with less variation and smaller predicted growing stock. The distributions of the second phase plots within the entire Tanzania is presented in Fig. 2. Ten plots per cluster were used for each stratum (Fig. 3). The distance between plot centers within a cluster was 250 m, which translates to 1280 m cluster lengths in east–west and north–south directions.

**Fig. 4:**
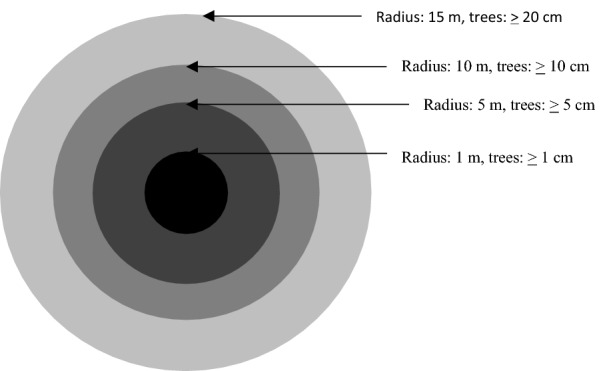
Layout of concentric sample plot

Plot cluster design aimed to increase efficiency during the forest inventory [16]. The sample plots were nested whereby they included 1, 5, 10 and 15 m radius concentric plots (Fig. 4). In uneven-aged natural forest stands, the number of small trees is much higher than the number of large trees. Therefore, to make the sampling efficient, small trees were measured within small sub-plots and large trees were measured within large sub-plots.

### Data collection

Within each plot, trees were measured for diameter at breast height (dbh, 1.3 m above soil surface) using caliper and identified for species [16]. The minimum dbh of trees measured within each nested concentric circle are specified in Fig. 4. Within 15 m radius, dead woods (≥ 5 cm) were also measured for length and diameter at their terminal ends. The plot size considered were of 15 m radius (0.07 ha) and minimum diameter stem was 5 cm until 14^th^ May 2011 when adjustments were made to minimum diameter of 10 cm and plot radius of 10 m (0.031 ha) in order to improve data collection speed. For all sample plots, slope was recorded. Plot radius was corrected for slope when slope exceeded 5%.

### Land cover sub-classes expansion factors

Estimation of carbon stock was based on plot layout design of the NFI described earlier. Accordingly, it was necessary to calculate Expansion Factor (*ExF*) for each respective stratum since simple mean of carbon stock would ignore the nature of the sampling design upon which the data were collected. The *ExF* describes the area in which a sample plot represents in each stratum. Since first phase sampling units were distributed proportionally to stratum area, the area of the stratum *p* (*A*_*p*_) was estimated as follows:

1 $${\hat{\text{A}}}\,{ = }\,{\text{A}}\, *\,\frac{{{\text{n}}_{\text{p}} }}{{{\text{n}}_{ 1} }}$$

where: $n_{p}$ is number of first phase plots in stratum *p* (ha); $n_{1}$ is total number of first phase plots; and $A$ is total inventory area (Mainland Tanzania area (ha)). Practical sequences of computation are shown below and further described in Tomppo, Malimbwi, Katila, Mäkisara, Henttonen, Chamuya, Zahabu and Otieno [13].

Plot area *ExF* of stratum *p* was computed as follows

2 $$ExF_{p} \, = \,\frac{{{\hat{\text{A}}}_{\text{p}} }}{{{\text{n}}_{\text{p}} }}$$

where: $\hat{A}_{p}$ is area of stratum *p*; and $n_{p}$ total number of plots observed in stratum *p*.

Consider $n_{k,p}$ number of plots of landcover sub-class *k* falling in stratum *p.* The area $\hat{A}_{kp}$ of landcover sub-class *k* in stratum *p* was computed as:

3 $${\hat{\text{A}}}_{\text{kp}} \, = \,\sum\limits_{k\varepsilon p} {{\text{n}}_{\text{k,p}} } \,*\,ExF_{P}$$

where: $n_{k,p}$ number of plots of land cover sub-class *k* in stratum *p;* and $ExF_{p}$ is Expansion Factor of stratum *p*.

Area of land cover sub-class *k* in the country is the summation of areas of land cover sub-classes *k* found in each stratum, i.e. $\varvec{\hat{A}_{k}} = \hat{A}_{k1} + \hat{A}_{k2} + \hat{A}_{k3} + \ldots \ldots \hat{A}_{kp}$ where *kp* is land cover sub-class *k* in stratum *p*.

### Estimation of tree and deadwood carbon values

#### Tree AGB and BGB

The AGB and BGB values of each measured tree in sample plot were estimated directly using appropriate allometric biomass models presented in Malimbwi et al. [17], URT [18] and tabulated further in Appendix 1. The models are based on the sample trees covering different land cover types in Mainland Tanzania. The plot levels values were then scaled up to per hectare level. Biomass models for few land cover types such as Open land: Salt crusts, Water: Inland water and tree species such as *Dalbergia melanoxylon*, *Grevillea robusta*, and *Eucalyptus* spp., were lacking. In such cases, volume models were used to compute volume which was then converted to biomass using appropriate species-specific wood density from the Global Wood Density database [19] and expansion factor of 1.4. For cases where species-specific wood density values were missing from the database, a default wood density value of 500 kg m^−3^ [9] was applied. BGB for some species including. *Adansonia digitata*, *Grevillea robusta*, *Eucalyptus* spp., *Dalbergia melanoxylon*, *Anacardium occidentale* and others could not be estimated directly using appropriate allometric BGB models. For such species BGB was estimated indirectly using a root to shoot ratio of 0.25 [20].

#### Dwb

DWB was estimated as the product of volume and specific wood density. Volume was computed using Smalian formula. The same procedures for conversion of tree volume to biomass were applied in conversion of dead wood volume to DWB although this did not involve the use of expansion factor. Irrespective of species, a wood density reduction factor of 0.97 was used for solid woods and 0.45 was used for the more decayed wood [10].

### Expansion of Tree AGB, BGB and DWB to plot level

Expansion of tree AGB and BGB; and DWB to plot level considered the concentric plot design (Fig. 4).

AGB, BGB and DWB plot values were obtained using the Eq. 4:

4 $$Y_{j } = \frac{{\mathop \sum \nolimits_{k\varepsilon j} \hat{Y}_{i} }}{{a_{i} }}$$

where $Y_{j }$ is AGB, BGB or DWB per hectare of a plot *j*; $\hat{Y}_{i}$ is AGB, BGB or DWB of a tree *i* in plot *j*; and $a_{i}$ is inclusion area (area of sub-plot *s*) of tree *i* in plot *j*.

### Estimation of carbon pools values of land cover sub-classes

The carbon pool values for the different land cover sub-classes (Table 1) were computed from plot values obtained in previous section. To obtain these values, *ExF* were applied as shown in the following equation

5 $$\hat{Y}_{k} = \mathop \sum \limits_{j \in k} Y_{j } \times ExF_{p}$$

where *j* and *k* correspond to every plot *j* with land cover sub-class *k*; $ExF_{p}$ plot expansion factor of stratum *p* where plot *j* resides.

The mean AGB, BGB or DWB per hectare for the land cover sub-class *k* can be computed using Eq. 6 below:

6 $$\bar{Y}_{k} = {\raise0.7ex\hbox{${\hat{Y}_{k} }$} \!\mathord{\left/ {\vphantom {{\hat{Y}_{k} } {\hat{A}_{k} }}}\right.\kern-0pt} \!\lower0.7ex\hbox{${\hat{A}_{k} }$}}$$

where: $\bar{Y}_{k}$ is the mean AGB, BGB or deadwood per ha in landcover sub-class *k*; $\hat{Y}_{k}$ is the total AGB, BGB or DWB in land cover sub-class *k*; and $\hat{A}_{k}$ is total area of landcover sub-class *k*.

The AGB, BGB and DWB values were converted to aboveground carbon (AGC), belowground carbon (BGC) and Deadwood Carbon (DWC) by multiplying the biomass by the default IPCC carbon fraction value of 0.47 [10].

### Aggregation of carbon pools values from sub-classes to primary classes

The sub-classes for the primary land cover classes were further aggregated into primary land cover classes level (i.e. forest, non-forest and wetland). The carbon pools values of each primary land cover class were estimated as a mean of the land cover sub-class estimates, which were weighted by their corresponding areas. The carbon pool value of a given primary land cover class was therefore computed using Eq. (7).

7 $$Y_{M} = \frac{{\mathop \sum \nolimits_{k = 1}^{n} Y_{k} \times \hat{A}_{k} }}{{\mathop \sum \nolimits_{i = 1}^{n} \hat{A}_{k} }}$$

where: *Y*_*m*_ is the weighted estimate of AGC, BGC or DWC per hectare, *a* is the area of land cover sub-class *k*, *X* is AGC, BGC or DWC per ha of the land cover sub-class and *n* is the number of land cover sub-classes in the primary land cover class.

### Estimation of uncertainty for carbon stock

Firstly, variance (Ϭ^2^*)* for stratum *p* was first estimated followed by estimation of Standard Error (SE) of land cover sub-class *k* using the Eq. 8.

8 $$SE_{k} = \sqrt[2]{{\mathop \sum \nolimits \frac{{r_{p}^{2} \times SE_{p}^{2} }}{{n_{p} }}}}$$

where: $SE_{k}$ is Standard Error of land cover sub-class *k*; r_*p*_ is proportion of stratum area to total area of land cover sub-class *k*; SE_*p*_ Standard Error of stratum *p*; *n*_p_ number of sampling units for *p*^th^ stratum.

Confidence Interval (X) of the mean for a land cover sub-class *k* was computed using the Eq. 9.

9 $$X_{k} = SE_{k} \times t$$

where: $CI_{k}$ is Confidence Interval of the mean; $SE_{k}$ is the stratum Standard Error and *t* is value read from *t*-distribution table at 95% confidence level.

Estimation of uncertainty of the carbon stock in each primary land cover followed the procedure described in Eq. 3.2 of IPCC [10] (Eq. 10). Errors were weighted and propagated for parameters with the same units of measurement.

10 $$U_{total} \, = \,\frac{{\sqrt {(U_{1} \, \times \,X_{1} )^{2} \, + \,(U_{2} \, \times \,X_{2} )^{2} \, + \cdots + (U_{n} \, \times \,X_{n} )^{2} \,} \,}}{{\left| {X_{1} \, + \,X_{2} \, + \cdots \left. { + \,X_{n} } \right|} \right.}}$$

where:  *U*_*total*_= percentage uncertainty of the sum of quantities (half the 95% confidence interval, divided by the total (i.e. the mean) and expressed as a percentage). The term “uncertainty” is based on the 95% confidence interval. X_i_ and  *U*_i_= uncertainty quantity and the associated percentage uncertainties, respectively.

## Results

### Carbon stocks for different land cover types

The average carbon stocks for the three carbon pools (AGC (t C ha^−1^), BGC (t C ha^−1^) and DWC (t C ha^−1^)) for each of the Land cover sub-class are presented in (Table 2). The areas of the respective land cover sub-class before aggregation into the primary vegetation sub-class are also presented in Table 2. The average total carbon stocks for aggregated land cover sub-classes (vegetation types) were computed and presented in Table 3. The values of the land cover sub classes ranged from 12.40 to 78.8 t C ha^−1^. The highest value was observed in mangrove while the lowest value was for thicket and wetland. When aggregating the land cover sub classes into primary land cover, the total carbon stock values for the three pools were ranging from 4.28 t C ha-1 to 33.35 t C ha^−1^. Uncertainty analysis was also done in line with the IPCC requirements. Among the three primary vegetation classes, wetland had higher uncertainty values of the carbon stock estimates compared to forest and non-forest class.

**Table 2:** Area coverage and average carbon stock in three IPCC pools for different land cover classes

Primary land cover class	Land cover sub-class	Area (ha)	AGC (t C ha^−1^)	BGC (t C ha^−1^)	DWC (t C ha^−1^)	Total carbon (t C ha^−1^)
Forest	Forest: Plantation	543,025.2	20.0	4.8	0.4	25.2
Forest	Forest: Mangrove	158,404.6	34.3	32.9	11.7	78.9
Forest	Forest: Humid Montane	953,866.4	62.0	15.5	4.8	82.4
Forest	Forest: Lowland	1,663,340.3	43.7	10.9	3.4	58.0
Forest	Woodland: Closed (> 40%)	9,006,125.9	32.4	13.9	1.6	47.8
Forest	Woodland: Open (10–40%)	36,219,223.5	20.0	8.8	1.1	29.9
Forest	Cultivated land (Wooded crops): Mixed tree cropping	148,306.2	18.1	4.5	2.1	24.7
Forest	Cultivated land: Wooded crops	1,493,112.2	10.9	3.5	0.7	15.1
Forest	Woodland: Scattered crop woodland (unspecified density) (wooded crops)	2,518,260.2	9.0	4.2	0. 8	14.0
Forest	Bushland: Thicket	951,895.3	6.3	1.578	0.5	8.4
Forest	Bushland: Thicket with emergent trees	303,396.0	14.7	8.1	2.2	25.0
Non forest	Bushland: Dense	1,961,501.5	9.0	2.2	0.6	11.8
Non forest	Bushland: Emergent trees	320,526.0	9.3	5.1	1.0	15.4
Non forest	Bushland: Open	2,796,873.4	10.0	4.0	0.2	14.3
Non forest	Bushland: Scattered cultivation	1,139,605.7	7.1	4.5	0.9	12.5
Non forest	Cultivated land: Agro-forestry system	1,352,158.0	4.0	1.0	0.5	5.5
Non forest	Cultivated land: Grain crops	9,748,899.9	2.5	0.6	0.2	3.3
Non forest	Cultivated land: Herbaceous crops	4,971,302.0	2.4	0.6	0.7	3.6
Non forest	Grassland: Bushed	433,078.4	3.3	1.4	0.1	4.8
Non forest	Grassland: Open	3,115,798.5	0.3	0.1	0.03	0.3
Non forest	Grassland: Scattered cropland	582,387.0	1.5	0.8	0.4	2.7
Non forest	Grassland: Wooded	4,667,016.0	4.8	2.2	0.2	7.2
Non forest	Not classified	4903.9	3.4	0.8	0.0	4.2
Non forest	Open land: Bare soil	159,354.4	2.0	0.5	1.2	3.7
Non forest	Open land: Rock outcrops	94,368.6	4.8	1.2	0.1	6.0
Non forest	Open land: Salt crusts	17,807.6	1.6	0.4	0.0	2.0
Non forest	Other areas	1,861,784.0	5.4	1.3	0.5	7.2
wetland	Water: Inland water	149,490.0	4.9	1.2	1.9	7.9
wetland	Water: Swamp	998,490.1	2.7	0.7	0.3	3.7

**Table 3:** Average total carbon stocks and uncertainties for different aggregated land cover sub-classes

Land cover sub-classes	Carbon (t C ha^−1^)	Uncertainty (%)
Forest: Closed woodland (> 40%)	47.82	0.62
Forest: Open woodland (10–40%)	29.93	1.24
Forest: Plantation	25.19	1.44
Forest: Mangrove	78.86	0.78
Forest: Montane and lowland	66.90	1.56
Forest: Thickets	12.40	1.34
Forest: Wooded crops	14.77	4.34
Overall for forest	*33.35*	*0.93*
Wetland	*4.28*	*11.3*
Non-forest	*5.81*	*1.8*

## Discussion

The overall objective of this paper was to compute carbon stocks for different land cover types of Mainland Tanzania using NFI (i.e. NAFORMA) data, which was conducted over a 5-year period of 2009 and 2014 by the Tanzania Forest Agency.

NAFORMA field plots cover all the land categories, making assessment of the areas and area change of all IPCC land categories and carbon pools possible. In this study carbon estimates have been reported for three IPCC carbon pools which included, above ground, below ground and deadwood. These pools are selected because, data have been collected on them through ground surveys as part of NAFORMA and, importantly, they are considered to represent the most important IPCC carbon pools for REDD+ reporting purposes. Based on this, total carbon was computed based on the three IPCC carbon pools as presented in Tables 2 and 3. Of all the land cover sub-classes, mangrove, lowland and humid montane forests had relatively higher total carbon estimates compared to other categories. This may be attributed by the presence of large trees in terms of dbh and height as compared to other land cover sub categories (Table 2). According to Brown et al. [21], large size trees tend to account for a large proportion of the AGB in mature forests; often between 30 and 40% of the AGB can be found in trees with diameters greater than 70 cm. On the other hand, lower values in other categories such as savanna may be attributed by the nature of the tree species as well as exposure different threats such as fire and selective logging. Generally, the carbon estimates presented for different categories reflect the carbon estimates values of the specific vegetation type for the precision intended at the national scale. There might be small deviations with the previous results reported in MNRT [9]. Given that the same dataset was used previously and reported in MNRT (2015) as well as the current study, the results presented in this paper are different from those reported previously in MNRT [9]. The noted differences may be attributed to the use of newly developed allometric models [See 17, 18], which are National and land cover specific. Specific vegetation type allometric models and direct biomass estimation approach are likely to be less biased and have smaller residual standard errors [22, 23] which may reduce error propagation at different scale of forest inventory.

In the previous reports, e.g. [9] estimation of biomass was done by indirect method as the product of wood density and tree stem volume. This was attributed by the lack of allometric models which were under construction at that time [see 13]. According to Njana [24] the use of indirect methods may results into large uncertainties of AGB estimates compared to the use tree allometric models. Thus the values presented in this paper may be highly relevant for REDD+ reporting as compared to the values reported in MNRT [9] and may be of interest for understanding the forest resources of Tanzania and their distribution across different land cover types at the National Scales. Results reported in our paper may also be different from reported case studies, such differences are attributable to scale (sub-national and national) and sampling design employed. However, if sampling employed is appropriate the margin of differences in estimates is not very large. There might be variations also in carbon estimates which results from the differences in the sampling design. As previously mentioned NAFORMA data was intended for generating the estimates of forest attributes at the national level precision, and thus may not capture much of the local variability presented by other studies. But since REDD+ reporting so far is currently aimed at the National scales the NAFORMA estimates are more appropriate. However, if NAFORMA has to be used for small area estimations, options for enhancing the precision using auxiliary remote sensing data will be the obvious choice [13]. Such approach is widely accepted as the best way of using NFI data for local based reporting [see 25–27].

Aggregation of the Land cover sub-classes to a more homogeneous classes was done to have larger number of sample units per respective category but also to be in line with categories which are used for reporting activity data in the country. Carbon stocks for respective aggregated land cover sub-classes are presented in Table 3. Mangrove seemed to have large values of average total carbon stock compared to other categories, this could be explained by large amount DWC in mangrove forests. The overall average carbon stock for the three major categories, i.e. forest, wetland and non-forest are less than the IPCC default values which range from 56 to 200 t C ha^−1^ for AGC. Most of the values reported in our paper fall below the lower-end of this range however the country specific estimates are considered to be more accurate and more appropriate to use. Thus in the context of IPCC levels of methodology, the reporting values of carbon stocks will support the country to report carbon emission at Tier 3 which essentially uses country-specific data (i.e. National inventory and allometric biomass models). The reported estimates also serve the purpose of forest carbon monitoring, either as an initial inventory of stocks from which changes can be estimated based on knowledge of effects of different factors such as harvesting and natural disturbances, or as a direct estimate of stock change from repeated inventories.

Uncertainty estimates are an essential element of a complete inventory of greenhouse gas emissions and removals. They should be derived for both the national level and the trend estimate, as well as for the component parts such as emission factors, activity data and other estimation parameters for each category. In this study we presented analysis of uncertainty which are within the bound of our expectations. There is high uncertainty in wetland, given the high variability of carbon stock in this category but also may be attributed by small number of field plots on this category as compared to forest and non-forest categories. However, generally the values of uncertainties for the three classes are within the reasonable ranges. As such they can be used for accounting the uncertainty for carbon emission factors elsewhere but also for computing overall uncertainty of carbon emission in construction of FREL for REDD+.

## Conclusion

In this paper, we have demonstrated that NFI data can be used for estimation of carbon stock for different land cover types. The study also demonstrate carbon stored in non-forestland. Non-forestland represents a land cover remaining after deforestation according to the national forest definition. Mangrove, lowland and humid montane forests store large quantities of carbon per unit area, if such forestlands are converted to non-forestland they lead into large emissions. Equally, in addition to large area occupied, woodlands also store large quantities of carbon. For the purpose of reducing emissions from deforestation and by considering national circumstances, all land covers should be managed although the management intensity and priorities should consider the significance emissions and other utilities such as biodiversity. The values presented in this paper correspond to IPCC tier 3 and can be used for estimation of land cover specific emission factors for and subsequently use emission factors to derive FREL for REDD+. However, if local based estimates values are needed, use of auxiliary data to enhance the precision of the area of interest should be considered.

## Appendix 1: Allometric AGB, volume and BGB models for different land cover classes in Mainland Tanzania

### Appendix 1a

See Table 4.

**Table 4:** Allometric AGB models for different land cover classes in Mainland Tanzania

Land cover sub-class	Species	AGB	Source
Forest: Humid Montane	All	0.3571 × dbh^1.744^ × ht^0.4713^	[28]
Forest: Lowland	All	0.3571 × dbh^1.744^ × ht^0.4713^	[28]
Forest: Mangrove	*Avicenia marina*	0.25128 × dbh^2.24351^	[29]
	*Soneratia alba*	0.25128 × dbh^2.21727^	[29]
	*Rhizophora mucronata*	0.25128 × dbh^2.26026^	[29]
	Others	0.19633 × dbh^2.010853^ × ht^0.29654^	[29]
Forest: Plantation	*Tectona grandis*	0.1711 × dbh^2.0047^ × ht^0.3767^	[30]
	*Pinus patula*	0.0550 × dbh^2.5968^	[31]
Woodland: Closed (> 40%)	All	0.0763 × dbh^2.2046^ × ht^0.4918^	[23]
	Baobab	2.234966 × dbh^1.43543^	[32]
Woodland: Open (10–40%)	All	0.0763 × dbh^2.2046^ × ht^0.4918^	[23]
	Baobab	2.234966 × dbh^1.43543^	[32]
Bushland: Thicket, dense	*Pseudoprosopis fischeri*	0.4276 × dbh^2.4053^ st^0.5290^	[33]
	*Combretum celastroides*	0.7269 × dbh^2.6710^ × ht^0.5737^ st^0.2039^	[33]
	Baobab	2.234966 × dbh^1.43543^	[32]
Bushland: Emergent trees	All	1.2013 × dbh^1.5076^	[33]
Bushland: Thicket with emergent trees	All	1.2013 × dbh^1.5076^	[33]
Bushland: Open	Others	0.0763 × dbh^2.2046^ × ht^0.4918^	[23]
	*Acacia and Commiphora* spp.	0.0292 × dbh^2.0647^ × ht^1.0146^	[34]
Grassland: Wooded	Others	0.0763 × dbh^2.2046^ × ht^0.4918^	[23]
	*Acacia and Commiphora* spp.	0.0292 × dbh^2.0647^ × ht^1.0146^	[34]
	Baobab	2.234966 × dbh^1.43543^	[32]
Grassland: Bushed Grassland: Open	Others	0.0763 × dbh^2.2046^ × ht^0.4918^	[23]
	*Acacia and Commiphora* spp.	0.0292 × dbh^2.0647^ × ht^1.0146^	[34]
	Baobab	2.234966 × dbh^1.43543^	[32]
Woodland: Scattered cropland (Unspecified density)	All	0.0763 × dbh^2.2046^ × ht^0.4918^	[23]
	Baobab	2.234966 × dbh^1.43543^	[32]
Bushland: Scattered cultivation	All	1.2013 × dbh^1.5076^	[33]
	Baobab	2.234966 × dbh^1.43543^	[33]
Grassland: Scattered cropland	All	1.2013 × dbh^1.5076^	[33]
Cultivated land: Agro-forestry system	All	0.051 * (dbh^2^ * ht)^0.93^	[35]
Cultivated land: Wooded crops	Coconut trees	3.7964 × ht^1.8130^	[36]
	Cashew nut trees	0.3152 × dbh^1.7722^ht^0.5003^	[37]
	Others	0.0763 × dbh^2.2046^ × ht^0.4918^	[23]
Cultivated land: Herbaceous crops	All	0.051 * (dbh^2^ * ht)^0.93^	[35]
Cultivated land: Mixed tree cropping	All	0.051 * (dbh^2^ * ht)^0.93^	[35]
Cultivated land: Grain crops	All	0.051 * (dbh^2^ * ht)^0.93^	[35]

### Appendix 1b

See Table 5.

**Table 5:** Allometric volume models for different land cover classes in Mainland Tanzania

Land cover sub-class	Species	Volume (m^3^)	Source
Forest: Plantation	*Eucalyptus* spp.	0.000065 × dbh^1.633^ht^1.137^	[38]
	*Grevillea robusta*	0.000065 × dbh^1.633^ht^1.137^	[38]
	*Others*	0.5 × 3.14 × (0.01 × dbh/2)^2^ × ht	[39]
Woodland: Closed (> 40%)	*Dalbergia melanoxylon*	0.00023 × dbh^2.231^	Malimbwi 2000
Woodland: Open (10–40%)	*Dalbergia melanoxylon*	0.00023 × dbh^2.231^	Malimbwi 2000
Open land: Bare soil	All	0.5 × 3.14 × (0.01 × dbh/2)^2^ × ht	[39]
Open land: Salt crusts	All	0.5 × 3.14 × (0.01 × dbh/2)^2^ × ht	[39]
Open land: Rock outcrops	All	0.5 × 3.14 × (0.01 × dbh/2)^2^ × ht	[39]
Water: Inland water	All	0.5 × 3.14 × (0.01 × dbh/2)^2^ × ht	[39]
Water: Swamp	All	0.5 × 3.14 × (0.01 × dbh/2)^2^ × ht	[39]
Other areas	All	0.5 × 3.14 × (0.01 × dbh/2)^2^ × ht	[39]

### Appendix 1c

See Table 6.

**Table 6:** Allometric BGB models for different land cover classes in Mainland Tanzania

Land cover sub-class	Species	BGB	Source
Forest: Mangrove	*Avicenia marina*	1.42040 × dbh^1.44260^	[29]
	*Soneratia alba*	1.42040 × dbh^1.65760^	[29]
	*Rhizophora mucronata*	1.42040 × dbh^1.68979^	[29]
	Others	1.42040 × dbh^1.59666^	[29]
Forest: Plantation	*Tectona grandis*	0.0279 × dbh^1.7430^ × ht^0.7689^	[30]
	*Pinus patula*	0.0027 × dbh^3.0579^	[31]
Woodland: Closed (> 40%)	All	0.1766 × dbh^1.7844^ht^0.3434^	[23]
	Baobab	AGB × 0.25	[32]
	*Dalbergia melanoxylon*	AGB × 0.25	[9]
Woodland: Open (10–40%)	All	0.1766 × dbh^1.7844^ht^0.3434^	[23]
Bushland: Thicket, dense	*Pseudoprosopis fischeri*	0.1442 × dbh^4.1534^ st^0.4117^	[33]
	*Combretum celastroides*	0.1006 × dbh^4.0062^ st^0.33499^	[33]
Bushland: Emergent trees	All	1.3803 × dbh^1.1671^	[33]
Bushland: Thicket with emergent trees	All	1.3803 × dbh^1.1671^	[33]
Bushland: Open	Others	0.1766 × dbh^1.7844^ht^0.3434^	[23]
	*Acacia and Commiphora* spp.	0.0593 × dbh^1.4481^ × ht^1.0210^	[34]
Grassland: Wooded	Others	0.1766 × dbh^1.7844^ht^0.3434^	[23]
	*Acacia and Commiphora* spp.	0.0593 × dbh^1.4481^ × ht^1.0210^	[34]
Grassland: Bushed			
Grassland: Open	Others	0.1766 × dbh^1.7844^ht^0.3434^	[23]
	*Acacia and Commiphora* spp.	0.0593 × dbh^1.4481^ × ht^1.0210^	[34]
Woodland: Scattered cropland (Unspecified density)	All	0.1766 × dbh^1.7844^ht^0.3434^	[23]
Bushland: Scattered cultivation	All	1.3803 × dbh^1.1671^	[33]
	Baobab	AGB × 0.25	[33]
Grassland: Scattered cropland	All	1.3803 × dbh^1.1671^	[33]
Cultivated land: Wooded crops	Coconuts trees	13.5961 × ht^0.6635^	[36]
	Others	0.1766 × dbh^1.7844^ht^0.3434^	[23]

## Abbreviations

REDD+: reducing emissions from deforestation and forest degradation
FREL: forest reference emission level
NFI: national forest inventory
NAFORMA: National Forest Resources Monitoring and Assessment
AGB: above ground biomass
BGB: below ground biomass
DWB: dead wood
